# The homology and function of the lung plates in extant and fossil coelacanths

**DOI:** 10.1038/s41598-017-09327-6

**Published:** 2017-08-23

**Authors:** Camila Cupello, François J. Meunier, Marc Herbin, Philippe Janvier, Gaël Clément, Paulo M. Brito

**Affiliations:** 1 Departamento de Zoologia, Universidade do Estado do Rio de Janeiro, R. São Francisco Xavier, 524-Maracanã, Rio de Janeiro, 20550–900 Brazil; 2 UMR 7208 (CNRS–IRD–MNHN–UPMC) Biologie des Organismes et Ecosystèmes Aquatiques, Département Adaptations du Vivant, Muséum national d’Histoire naturelle, CP026, 43 rue Cuvier, Paris, 75231 France; 3 UMR 7179 (CNRS–MNHN) Mécanismes Adaptatifs des Organismes aux Communautés, Département Adaptations du Vivant, Muséum national d’Histoire naturelle, 57 rue Cuvier, Paris, 75231 France; 4 UMR 7207 (Sorbonne Universités–MNHN–CNRS–UPMC/Paris6) Centre de Recherche sur la Paléobiodiversité et les Paléoenvironnements, Département Origines & Evolution, Muséum national d’Histoire naturelle, 57 rue Cuvier, CP38, Paris, F-75005 France

## Abstract

The presence of a pulmonary organ that is entirely covered by true bone tissue and fills most of the abdominal cavity is hitherto unique to fossil actinistians. Although small hard plates have been recently reported in the lung of the extant coelacanth *Latimeria chalumnae*, the homology between these hard structures in fossil and extant forms remained to be demonstrated. Here, we resolve this question by reporting the presence of a similar histological pattern–true cellular bone with star-shaped osteocytes, and a globular mineralisation with radiating arrangement–in the lung plates of two fossil coelacanths (*Swenzia latimerae* and *Axelrodichthys araripensis*) and the plates that surround the lung of the most extensively studied extant coelacanth species, *L. chalumnae*. The point-for-point structural similarity of the plates in extant and fossil coelacanths supports their probable homology and, consequently, that of the organ they surround. Thus, this evidence questions the previous interpretations of the fatty organ as a component of the pulmonary complex of *Latimeria*.

## Introduction

An elongated organ comprising ossified plates was first reported during the 19^th^ century in the abdominal cavity of almost all Palaeozoic and Mesozoic coelacanth families^1–5^, including Hadronectoridae, Rhabdodermatidae, Laugiidae, Whiteiidae, Mawsoniidae, and Latimeriidae. However, small lung plates have been identified only recently in the extant coelacanth *Latimeria chalumnae* ^6^, but the homology between the lung plates of extant and fossil coelacanths remained to be proven, as did that between the pulmonary complex of fossil and extant coelacanths.

Bone, which is the main component of osteichthyans skeleton, essentially has three components: bone cells, extracellular fibrillary organic material or organic matrix, and extracellular mineral material^7, 8^. Although the presence of mineralised tissue in the anterior part of the gas bladder has been reported in some ophidiiform^9–12^ and perciform teleosts^13^, such a calcified organ comprising true cellular bone tissue^5, 14^ and occupying most of the abdominal cavity is a unique feature of coelacanthiform actinistians.

Here, we describe the similar structures of extant and fossil coelacanth lung plates, which demonstrate that the large, long enigmatic calcified organ of many Palaeozoic and Mesozoic coelacanths show the same structure as the peculiar, minute, hard plates that surround the vestigial lung of the iconic extant coelacanth *L. chalumnae*. Our results suggest that the lung plates of fossil and extant coelacanths are homologous^6^ and, consequently, that the large calcified organ of fossil coelacanths is homologous to the vestigial lung seen in the extant species *L. chalumnae*. These bony plates probably functioned as volume regulators and protected against hydrostatic pressure.

## Results

### Lung plates morphology

**Figure 1:**
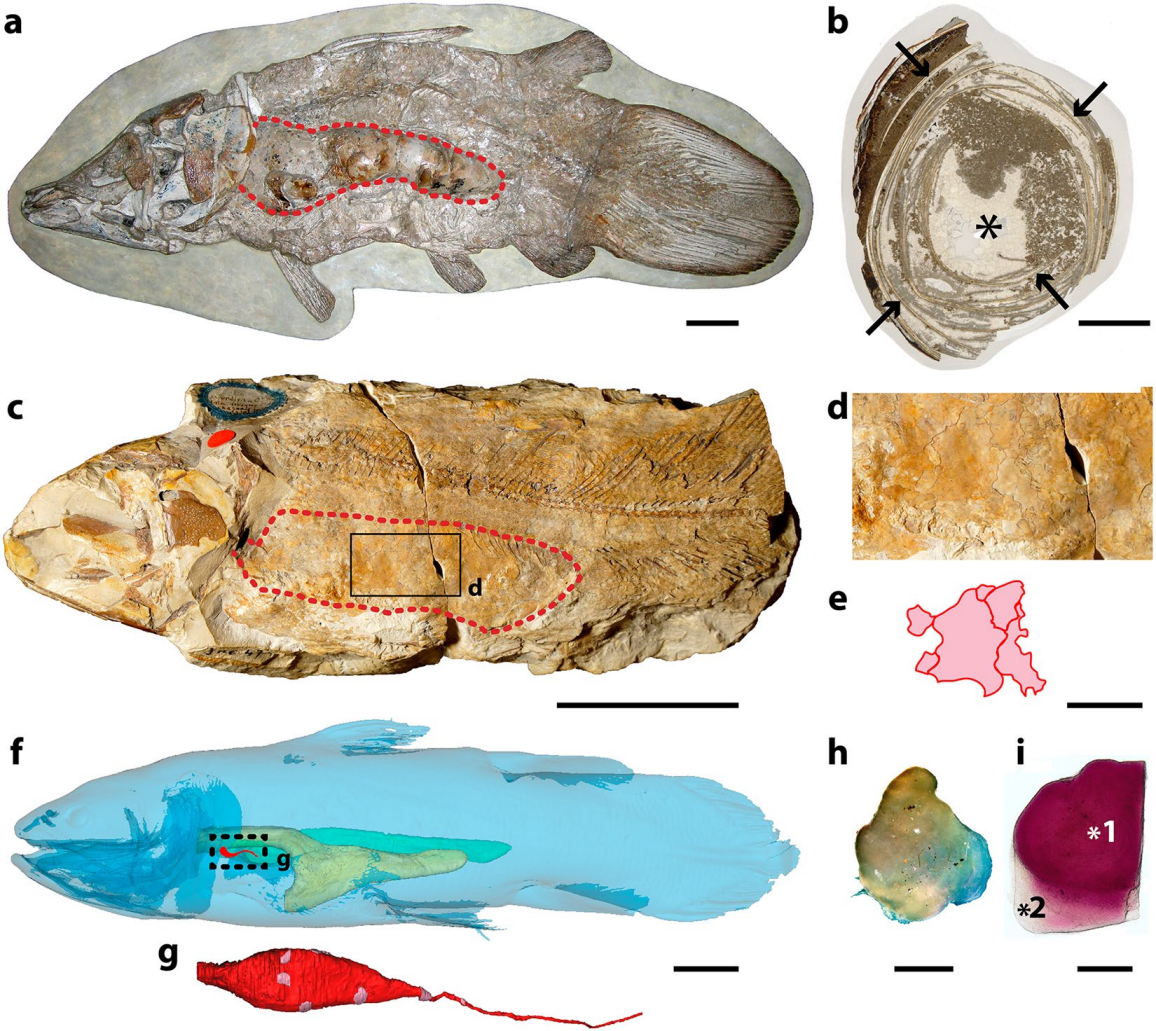
Lung plates of fossil and extant coelacanths. (**a**) Mirrored image of *Axelrodichthys araripensis* (KMNH VP 100,328), Lower Cretaceous of the Santana Formation, Araripe Basin, Brazil (Photo: Y. Yabumoto). (**b**) Transverse ground cross-thin section of an uncrushed lung of *Axelrodichthys araripensis* (‘Josa collection’); arrows indicating ossified plates and asterisk pointing to lung lumen. (**c**) *Swenzia latimerae* (Holotype, MNHN.F.JRE 47), Upper Oxfordian of Burgundy, France. Specimen in left lateral view. (**d**) Close-up of boxed area in (**c**), focusing on lung covered by ossified plates. (**e**) Interpretive drawing of ossified lung plates of *Swenzia latimerae* lung in (**c**) and (**d**). (**f**) 3D reconstruction of *Latimeria chalumnae*, left lateral view of adult specimen CCC 22 (130 cm TL). (**g**) Three-dimensional reconstruction of vestigial lung and bony plates of adult specimen CCC 28. (**h**) Isolated hard plate of *Latimeria chalumnae*, adult specimen CCC 24. (**i**) Isolated half of a hard plate of *Latimeria chalumnae*, adult specimen CCC 79 stained with alizarin red; asterisk 1, mineralised portion of plate stained dense pink; asterisk 2, extremity of plate, corresponding to non-mineralised region. Green, fatty organ; pink, ossified plates; red dashed line, outline of calcified lung; red, lung; yellow, oesophagus and stomach. Scale bars, 5 cm (**a**,**c**) 1 cm (**b**,**d**,**e**,**g**) 10 cm (**f**) 0.25 cm (**h**) 0.05 cm (**i**).

**Figure 2:**
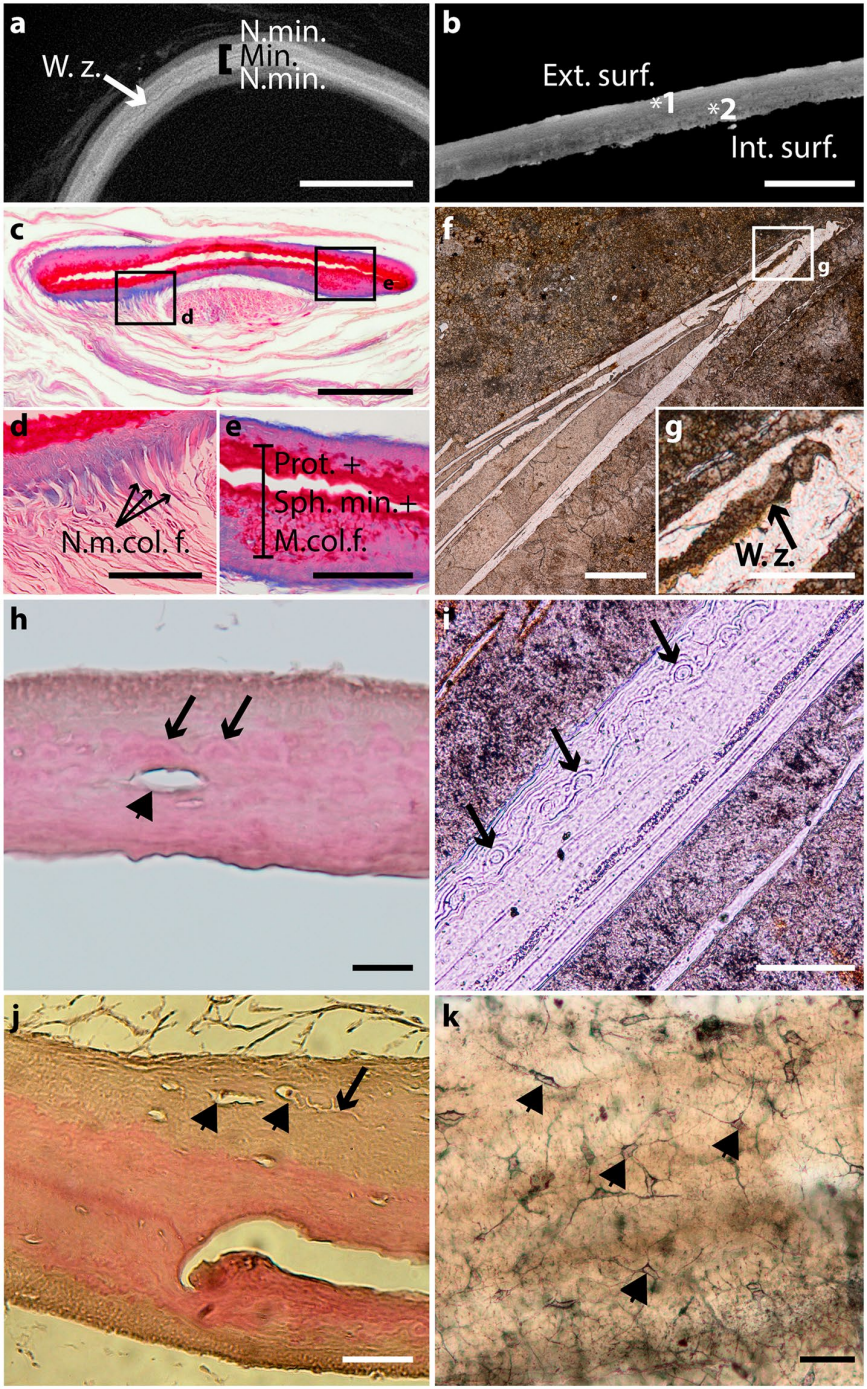
Extant and fossil coelacanth lung plates. (**a**) Virtual transverse section of an ossified plate of the extant coelacanth *Latimeria chalumnae* (adult specimen CCC 24). (**b**) Virtual section of an ossified plate of the fossil coelacanth *Axelrodichthys araripensis* from the Lower Cretaceous of the Santana Formation, Araripe Basin, Brazil (‘Josa collection’ MNHN, Paris); asterisk 1, external surface; asterisk 2, internal surface. (**c**) Histological thin section of a *Latimeria chalumnae* lung plate (adult specimen CCC 5, azocarmin coloration). (**d**) Close-up of boxed area in (**c**), focussing on collagen fibres at the extremity of the plate, which corresponds to the non-mineralised region. (**e**) Close-up of boxed area in (**c**), focussing on mineralised portion of the plate comprising proteoglycans, collagenous fibres, and spheritic mineralisation. (**f**) Ground cross-thin section of *Axelrodichthys araripensis* (UERJ-PMB 143) lung plate showing central artefactual fracture. (**g**) Close-up of boxed area in (**f**), focussing on weakness zone. (**h**) Histological thin section of a *Latimeria chalumnae* lung plate (haematoxylin/eosin coloration, adult specimen CCC 24); black arrows point to mineralised globules with a radiating arrangement; arrowhead points to osteocyte lacunae. (**i**) Ground cross-thin section of *Axelrodichthys araripensis* lung plate (UERJ-PMB 143); black arrows point to mineralised globules with a radiating arrangement. (**j**) Histological thin section of an ossified plate of the extant coelacanth *Latimeria chalumnae* (adult specimen CCC 24); arrowheads point to star-shaped osteocytes and arrow to canaliculi. (**k**) Ground cross-thin section of an *Axelrodichthys araripensis* lung plate (UERJ-PMB 143); arrowheads point to star-shaped osteocytes with ramified canaliculi for cytoplasmic processes. Ext. surf., external surface; Int. surf., internal surface; Min., mineralised portion; m. col. f., mineralised collagen fibres; n. m. col. f., non-mineralised collagen fibres; n. min., non-mineralised portion; prot, proteoglycans; sph. min., spheritic mineralisation; w. z., weakness zone. Scale bars, 500 µm (**a–c**,**f**) 100 µm (**d**,**e**,**i**) 2 µm (**g**) 20 µm (**h**) 50 µm (**j**,**k**).

**Figure 3:**
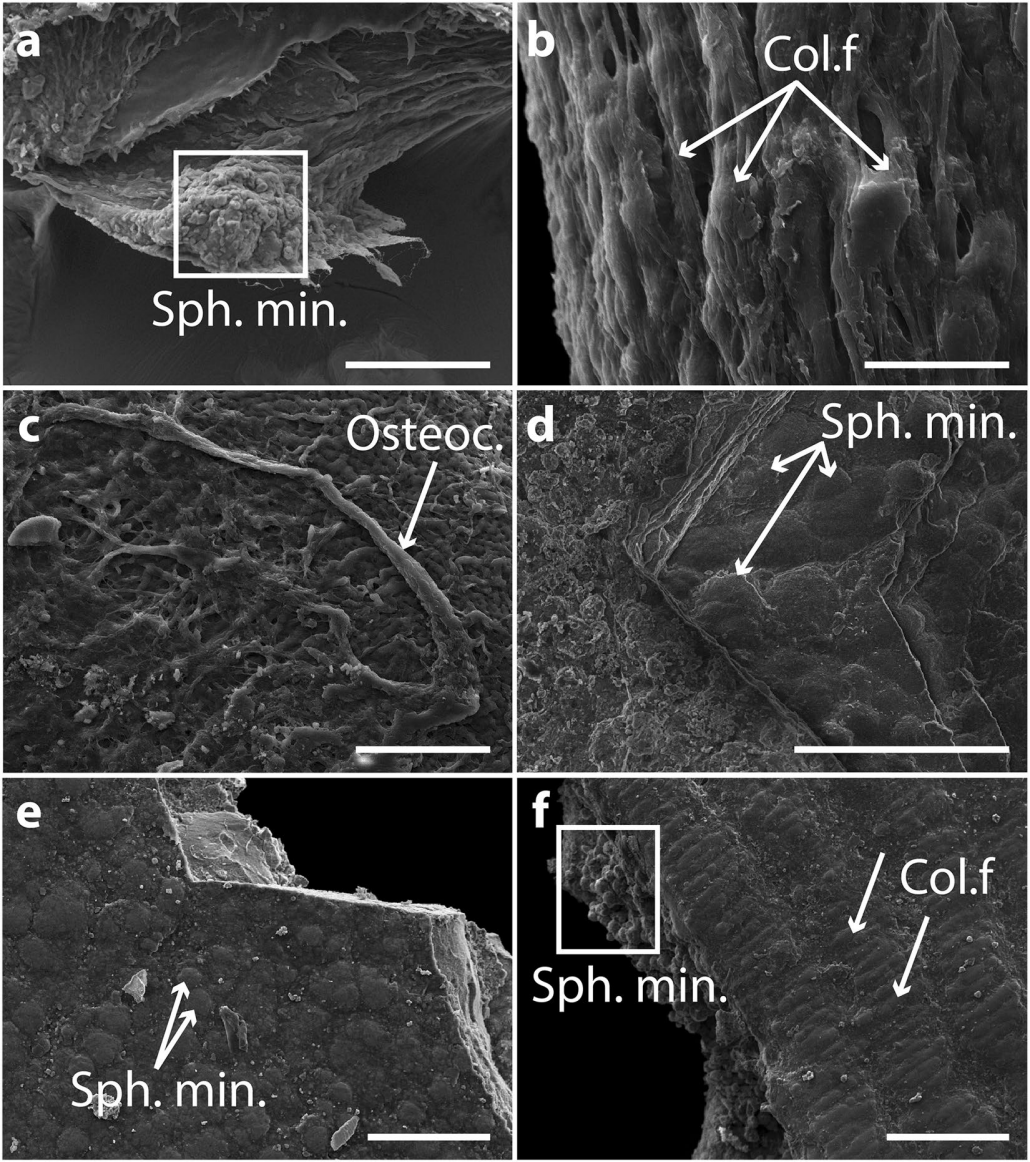
Scanning electron microscopy of extant and fossil coelacanth lung plates. (**a**) SEM image revealing the presence of spherules of spheritic mineralisation in a lung plate of *Latimeria chalumnae* (adult specimen CCC 24). Boxed area indicates spherules. (**b**) SEM image of a *Latimeria chalumnae* lung plate (adult specimen CCC 79) showing collagen fibres (white arrows). (**c**) SEM image of mineralised portion of a lung plate of *Latimeria chalumnae* (adult specimen CCC 79); white arrow indicates cytoplasmic processes departing from potential osteocytes. (**d**) SEM image of an *Axelrodichthys araripensis* lung plate (‘Josa collection’); white arrows indicate spherules of spheritic mineralisation. (**e**) SEM image of a *Swenzia latimerae* lung plate (MNHN.F.JRE 47); white arrows indicate presence of spherules characterizing spheritic mineralisation. (**f**) SEM image of a *Swenzia latimerae* lung plate (MNHN.F.JRE 47); boxed area indicates spherules characterizing spheritic mineralisation; white arrows indicate collagen fibres. Col. f., collagen fibres; sph. min., spheritic mineralisation; osteoc., osteocytes. Scale bars, 100 µm (**a**,**d**) 20 µm (**b**) 50 µm (**c**,**e**,**f**).

The abdominal cavity of most Palaeozoic and Mesozoic coelacanths houses a large space that is surrounded by variably shaped ossified plates that were termed ‘lung plates’ because they were assumed to cover the lung^5^ (Fig. 1a–e; Supplementary Fig. 1). Here, we compare them to the small hard plates that were recently observed around the lung of the living coelacanth *L. chalumnae* ^6^ (Fig. 1f–i), by using several different methods: (1) new partial dissections of adult specimens (n° CCC 3, CCC 5, CCC 24, CCC 28, CCC 79) (Fig. 1h,i); (2) virtual sections of computerized axial tomography scans and three-dimensional reconstruction of isolated lung plates and isolated viscus of the extant coelacanth *L. chalumnae* (specimens n° CCC 3, CCC 24, and CCC 28), and Cretaceous coelacanths *Axelrodichthys araripensis* (‘Josa collection’) and *Macropoma mantelli* (NHMUK PV P 2051) (Figs 1g and 2a,b; Supplementary Fig. 1); (3) histological thin sections of isolated lung plates of *L. chalumnae* (adult specimens n° CCC 5 and CCC 24) (Fig. 2c–e,h,j); (4) ground cross sections of *A. araripensis* (UERJ-PMB 143) (Fig. 2f,g,i,k); (5) scanning electron microscopy of isolated lung plates of Cretaceous *A. araripensis* (‘Josa collection’), Jurassic *Swenzia latimerae* (MNHN.F.JRE 47), and the extant coelacanth *L. chalumnae* (CCC 24 and CCC 79) (Fig. 3).

Due to the scarcity of *L. chalumnae* specimens in collections worldwide, particularly of specimens at early ontogenetic stages, it was not possible to dissect embryos and juveniles to check for the presence of lung plates. However, specimens at three embryonic stages and a juvenile have been analysed by using long propagation phase contrast synchrotron X-ray microtomographs^6^, but no plates were observed. Either the latter are lacking at these ontogenetic stages, or the technique used was unable to make them discernible on the virtual sections.

The lung plates of *L. chalumnae* are separated from each other and embedded in the conjunctive sheath that entirely covers the lung (Fig. 1g). The residual cord that prolongs the lung posteriorly only displays a few plates (Fig. 1g), and no plates are embedded in the multiple sheaths covering the fatty organ. The small, hard plates of the extant coelacanth lung present a variable general morphology, with an irregular rounded shape and no distinctive pattern (Fig. 1h,i). These plates have a concave face turned towards the lung, whereas the opposite face is convex, and their margins are thinner than their centre and weakly translucent.

To detect the presence of calcium in these plates and compare it with that of the ossified lung plates of fossil coelacanths, half of a plate was stained with alizarin red^15–17^. The obtained red coloration revealed the presence of calcium phosphate in the main part of the plate (Fig. 1i). Its margins, which correspond to a non-mineralised region comprising collagen fibres (see below), did not stain with alizarin red and retained a translucent aspect (Fig. 1i).

### Lung plates histology

Sections of isolated lung plates of the extant coelacanth *L. chalumnae* and of the fossil coelacanth *A. araripensis* revealed layers of primary cellular bone and osteocytes. In *L. chalumnae*, the deposition of bone matrix appears to proceed from the centre of the lung plates to the periphery (Fig. 2a,c–e,h,j), which can be structurally divided into three main regions: the internal region composed of a ground substance of proteoglycans, with seemingly weakness zone (Fig. 2c,e); the middle region made up of collagen fibres, spheritic mineralisation, and imbedded cells (Fig. 2e,h,j); and the non-mineralised external region composed of collagen fibres that appear to attach the plates to the lung’s conjunctive sheath (Fig. 2d).

The ossified lung plates of *L. chalumnae* and *A. araripensis* present a central weakness zone, usually with an indented pattern (Fig. 2a,c,e–g) that is revealed through the study of histological thin sections, ground sections, and scanning electron microscopy (SEM). In *L. chalumnae*, this central weakness zone displays a large gap (Fig. 2c,e) corresponding to a fracture in the middle of the proteoglycan region. Proteoglycans are related to collagen fibres and their degradation appears to be the prelude to collagenous extracellular matrix mineralisation^7^. The fracture of the weakness zone might be due to a preparation artefact of the histological thin sections because it is present in all histological thin sections prepared from both complete viscus and isolated lung plates. Moreover, the weakness zone is clearly present but has no internal gap on slices of high-resolution computerized axial tomography scans (Fig. 2a). The gap in the central zone of the lung plates of the fossil *A. araripensis*, which is considered here to be an enlargement of the weakness zone, may be due to post-mortem degradation and compaction during fossilisation, regardless of whether the layers are separated by the limestone matrix (Fig. 2f,g).

The mineralised portions of fossil and extant coelacanth lung plates comprise osteocytes (Fig. 2h,j,k), collagen fibres and spherules (Fig. 2d,e,h,i). This spheritic mineralisation is characterized by the formation of mineralised globules with a radiating arrangement^7, 18, 19^. In adult specimens of *A. araripensis*, only the mineralising front is preserved. New histological thin-sections of *L. chalumnae* lung plates clearly show, for the first time, the presence of star-shaped osteocytes with many cytoplasmic processes that extend within the thickness of the plate (Fig. 2h,j). New ground cross-sections of *A. araripensis* lung plates were also prepared to highlight the presence of star-shaped osteocytes (Fig. 2k), which was described in a previous study^5^.

### Scanning electron microscopy of the lung plates

The mineralised portion of extant (*L. chalumnae*) and fossil (*A. araripensis* and *S. latimerae*) coelacanth lung plates, analysed by SEM imaging (Fig. 3), also reveal spherules of spheritic mineralisation in the centre of the plates (Fig. 3a,d–f). Those of *L. chalumnae* also show cytoplasmic processes arising from presumed osteocytes, embedded in the bone matrix (Fig. 3c).

SEM images of the non-mineralised portion of *L. chalumnae* and *S. latimerae* lung plates display a collagenic peripheral portion formed by packed microfibres (Fig. 3b,f); this was also described in juveniles of *A. araripensis*, which do not present ossified plates at this early developmental stage^5^. In *S. latimerae*, the collagenic portion has been already described as thin parallel striations of the ossified plates^4^. Whereas the lung plates of adult specimens of *S. latimerae* and *L. chalumnae* display successive collagenic strata intercalated with spheritic mineralisation, those of *A. araripensis* display only their ossified portion preserved.

## Discussion

The presence of a functional lung in fossil coelacanths is presumed based on the presence of an opened pneumatic duct (in extant and fossil coelacanths); the presence of a calcified organ in the abdominal cavity of almost all known fossil coelacanths (including *Latimeria*’s closest relatives e.g., *Swenzia*, *Macropoma*); the presence of a functional lung in all extant sarcopterygians; the ventral position of the vestigial lung (relative to the alimentary tract) in extant coelacanths; the normal development of *Latimeria* lung at the early embryonic stage; the poorly developed gills of *Latimeria* (with thick walls compared to its body mass); the spiracular chamber present in *Latimeria* (although not opened to the outside); and the different living environments of fossil coelacanths (most often in shallow water environments that may have been dysoxic)^5, 6, 20–22^.

The lung of the extant coelacanth *L. chalumnae* is covered with small, scattered plates and exhibits a vestigial state^6^, in contrast to the well-developed calcified organ surrounded by the ossified plates of fossil coelacanth taxa^5^ (Fig. 1). The hard plates of the fossil (*A. araripensis* and *S. latimerae*) and extant coelacanths are laminated, extended throughout lung length, constituted by true cellular bone with star-shaped osteocytes and globular mineralisation with a radiating arrangement (Figs 2 and 3). In addition, fossil and extant coelacanth lung plates also show a proteoglycan ‘central weakness zone’ with an indented pattern. This was previously misinterpreted through analysis of histological thin sections as the lumen of vestigial pulmonary arteries in *L. chalumnae* ^23^. Globular mineralisation is also known from the anterior part of the gas bladder in ophidiiform teleosts as so called ‘rocker-bone’. However, this structure does not comprise true bone tissue with osteocytes and it has a fibrillary matrix that is devoid of type I or II collagen fibres^12^. Furthermore, the rocker-bone is kidney-shaped, is restricted to the anterior portion of the gas bladder, and does not contain mineralised laminae^12^.

The presence of collagen fibres instead of true bone tissue in the lung plates of juveniles of fossil coelacanths^5^, in addition to the non-mineralised collagenic external portion of the extant coelacanth lung plates, suggests that collagen fibres are the prelude to the formation of true bone tissue in these structures. In the embryos and juvenile of *L. chalumnae* ^6^, lung plates appear to be absent or non-ossified.

The extensively developed lung plates that are present in adult fossil coelacanths may have been required for lung ventilation^5^. These plates most likely functioned in volumetric regulation and protection of the lung against hydrostatic pressure^5, 14^. In the extant coelacanth *L. chalumnae*, the lung plates appear to be attached to the lung conjunctive sheath by non-mineralised collagen fibres. This mode of attachment suggests the mobility of lung plates for volumetric variation during air-breathing, thereby providing a clue to the function of this structure in fossil coelacanths. This function was lost in recent coelacanths, which became adapted to deeper water conditions and therefore the lung became vestigial^6^.

Fossil and extant lungfishes do not show calcified lung in the abdominal cavity, and some have elongate ribs, such as the Palaeozoic *Howidipterus donnae*, *Barwickia downunda* ^24^, *Scaumenacia curta*, *Fleurantia denticulata*, and *Ctenodus allodens* ^25, 26^ and the Triassic *Paraceratodus germaini* ^27^. The reduction of pleural ribs in extant lungfishes appears to have occurred secondarily, because long pleural ribs are found in some Palaeozoic and Early Mesozoic lungfishes. Pleural ribs primarily have a protective function for the lung and viscera^28^. Although they are not the only structure that assists in air-breathing in fossil lungfishes, the presence of well-developed and curved pleural ribs may have favoured the evolution of lungs without ossified plates. Indeed, in most of fossil coelacanths with a calcified lung in the abdominal cavity, the ribs are reduced or absent, whereas some coelacanths that lack a calcified lung, such as *Diplurus*, display well-developed ribs^5, 29^.

Although the presence of lungs is presumed in early bony fishes^30^, the presence of this organ among fossil taxa is only evidenced in coelacanths^5, 6, 14^. In fossil lungfishes, air-breathing behaviour is mainly inferred from the presence of cranial ribs^28^, which may have had a function in air-gulping^28, 31^, and from the well-developed, and sometimes strongly curved, pleural ribs that are described as an adaptation to expanded lungs^24^.

The presence of ossified lung plates in extant coelacanths confirms the presence of a lung in many Mesozoic coelacanthiforms. Our data, based on specimens of *L. chalumnae* and adult specimens of three fossil taxa, suggest that fossil coelacanths once possessed functional lungs covered by functional ossified plates that likely acted as regulators of volume variation and protected against hydrostatic pressure. Since the coelacanth history can be traced back to the Early Devonian^32^, wider attention should be focused on the possible presence of lungs with ossified plates in early coelacanths.

## Methods

### Specimens

Six adult specimens of *L. chalumnae*, housed in the Collection of Comparative Anatomy of the Muséum national d’Histoire naturelle (MNHN, France), were analysed during the development of this study: CCC 3 (male, 129 cm TL, Comoro Islands, caught 1953), CCC 5 (male, 127 cm TL, Comoro Islands, caught 1954), CCC 22 (male, 130 cm TL, Comoro Islands, caught 1960), CCC 24 (female, 145 cm TL, Comoro Islands, caught 1960), CCC 28 (male, 130 cm TL, Comoro Islands, caught 1961), and CCC 79 (female, 163 cm TL, Comoro Islands, caught 1972). All adult specimens of *L. chalumnae* studied here provided evidence of small plates surrounding the vestigial lung. All specimens were fished in in the Comoro Islands between 1953 and 1972, prior to this project, and were stored in formalin (except CCC 79, which was stored in alcohol after a short fixation in formalin).

Among the fossil specimens, we analysed the plates of adult specimens of *S. latimerae* (MNHN.F.JRE 47) from the Upper Jurassic of Burgundy, France; *A. araripensis* (‘Josa collection’ and UERJ-PMB 143) from the Lower Cretaceous of Santana Formation, Araripe Basin, north-eastern Brazil; and *M. mantelli* (NHMUK PV P 2051) from the Upper Cretaceous of Chalk Formation, Lewes, Sussex, UK. The growth series of fossil coelacanths are very rare. A few juvenile specimens of *A. araripensis* are known, but their lung plates are barely calcified and hardly provide reliable information about the extent of the lung at such early stages. The fossil specimens are housed in the vertebrate palaeontology collection of the MNHN (Paris, France), at the Universidade do Estado do Rio de Janeiro (Rio de Janeiro, Brazil), and at the Natural History Museum (London, UK).

No special permission was required to publish results from the specimens deposited in these scientific collections.

### Experimental procedures

X-ray computed micro-tomography (µCT) scanning was performed at the AST-RX Platform of the Muséum national d’Histoire naturelle, Paris, for isolated lung plates of extant (*L. chalumnae* CCC 24) and fossil coelacanths (*A. araripensis* ‘Josa collection’), for an isolated viscus of *L. chalumnae* (CCC 28), and for the fossil coelacanth *M. mantelli* (NHMUK PV P 2051). For the lung plate of CCC 24 (transferred to alcohol and stained in 5% phosphomolybdic acid for 7 days^33, 34^), the scanning parameters were as follows: voltage 35 kV, current 350 mA, voxel size 2.8 µm and 1,800 views. For the fossil lung plate (*A. araripensis* ‘Josa collection’), the voltage was 55 kV, current 170 mA, voxel size 2.97 µm, and the views number was 2,000. For CCC 28, the parameters were as follows: voltage 80 kV, current 430 mA, voxel size 54.24 µm and 2,550 views. For NHMUK PV P 2051, the voltage was 145 kV, current 490 mA, voxel size 143.11 µm and the view number was 1,500. For the complete specimen CCC 22, a high-resolution CAT scan was performed in a Parisian hospital (France) using voltage 120 kV, current 158 mA, and voxel size 742 µm and the views number was 1,807. Phoenix datos|x 2.0 reconstruction software was used to reconstruct and export images into 16-bit TIFF stacks. Segmentation and three-dimensional rendering were performed at the Palaeontology Imaging Unit of the Département Histoire de la Terre/UMR 7207 CR2P CNRS/MNHN/UPMC and at the Laboratório de Ictiologia Tempo e Espaço of the Universidade do Estado do Rio de Janeiro using MIMICS Innovation Suite 16.0, 18.0, and 19.0 (Materialise).

An isolated lung plate from the adult specimen CCC 79 of *L. chalumnae* was cut in two equal parts. To verify the presence of calcium phosphate, the first semi-plate was stained with 1% alizarin red in KOH 0.5% solution (for the second semi-plate, see below). The plate was then rinsed in distilled water and dehydrated in alcohol before light microscopy examination.

Histological thin sections of the oesophagus and lung of the extant coelacanth *L. chalumnae* (adult specimen CCC 5) were prepared by Millot *et al*.^23^ and are part of the historical material of *L. chalumnae* housed in the Collection of Comparative Anatomy of the MNHN, for which no detailed protocol is archived. New histological thin sections of isolated plates of the adult specimen CCC 24 were also prepared using haematoxylin/eosin coloration. These plates were dehydrated, embedded in wax, and cut with a microtome. The material was analysed via light microscopy (natural transmitted light). Unstained sections with polarized light (Nomarsky) were also observed.

Histological structures of *A. araripensis* lung plates were described through new and previously studied ground sections of the adult specimens ‘Josa collection’ and UERJ-PMB 143^5^. Ground sections (1 cm) were cut from the lung of these specimens. Each slice was embedded in Stratyl (Chronolite 2060)^5^. Sections (cross and horizontal sections in relation to the antero-posterior axis of the lung) were cut with a saw, glued on a glass slide, ground to the appropriate thickness, and observed in transmitted natural and polarized light^5^.

For scanning electron microscopy, plates of two specimens of the extant coelacanth *L. chalumnae* (CCC 24 and CCC 79), and two fossil species (*A. araripensis* ‘Josa collection’ and *S. latimerae* MNHN.F.JRE 47) were sampled. The spatial organization of the collagenous network of the *Latimeria* plate was processed using a plate that had been frozen in liquid nitrogen; the sample was broken, dried and gold-coated for SEM. For the mineralising front, we used the second semi-plate. It was plunged in 1% KOH solution to remove the unmineralised organic matter, rinsed in distilled water, dehydrated, dried and gold-coated for SEM. SEM images presented in Fig. 3 were obtained using a JEOL benchtop SEM (JCM-6000).

### Data Availability

The protocols used in the development of this study are available in the ‘Experimental Procedures’ section.

## Electronic supplementary material

Supplementary figure 1
