# Supplementary material for: The homology and function of the lung plates in extant and fossil coelacanths
Source: Sci Rep. 2017 Aug 23;7:9244. doi: 10.1038/s41598-017-09327-6 (PMC5569016; doi:10.1038/s41598-017-09327-6)
Supplement: Supplementary file 1 — Supplementary figure 1 [file 41598_2017_9327_MOESM1_ESM.pdf]

The homology and function of the lung plates in extant and fossil coelacanths

Camila Cupello[1*], François J. Meunier[2], Marc Herbin[3], Philippe Janvier[4], Gaël Clément[4], Paulo M. Brito[1]

[1]Departamento de Zoologia, Universidade do Estado do Rio de Janeiro, R. São Francisco Xavier, 524-Maracanã, Rio de Janeiro 20550–900, Brazil. [2]UMR 7208 (CNRS–IRD–MNHN–UPMC) Biologie des Organismes et Ecosystèmes Aquatiques, Département Adaptations du Vivant, Muséum national d'Histoire naturelle, CP026, 43 rue Cuvier, Paris 75231, France. [3]UMR 7179 (CNRS–MNHN) Mécanismes Adaptatifs des Organismes aux Communautés, Département Adaptations du Vivant, Muséum national d'Histoire naturelle, 57 rue Cuvier, Paris 75231, France. [4]UMR 7207 (Sorbonne Universités–MNHN–CNRS–UPMC/Paris6) Centre de Recherche sur la Paléobiodiversité et les Paléoenvironnements, Département Origines & Evolution, Muséum national d'Histoire naturelle, 57 rue Cuvier, CP38, Paris F-75005, France.

*Correspondence should be addressed to C.C. (camila.dc@gmail.com)

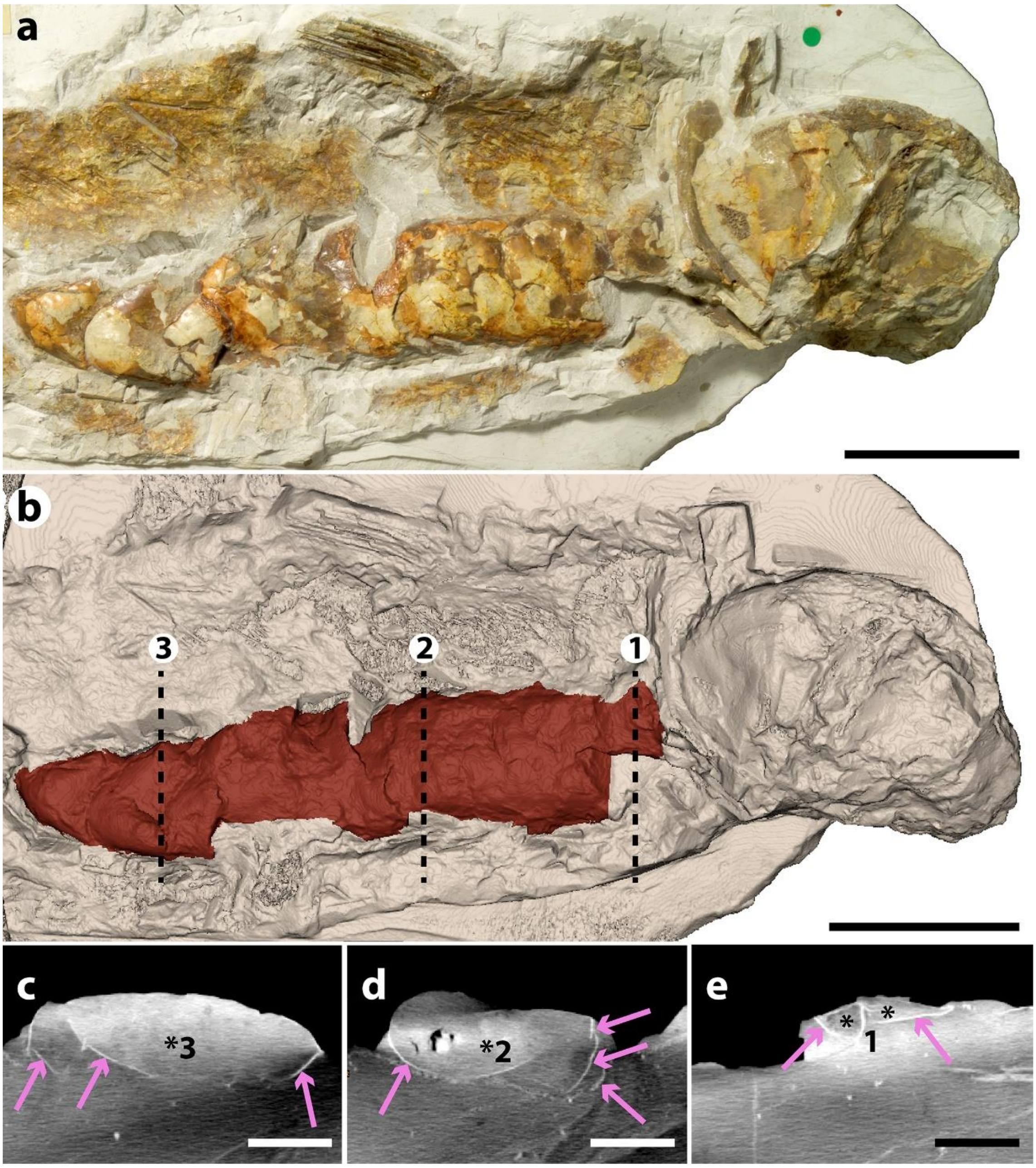

Supplementary figure 1| The calcified lung of *Macropoma mantelli* NHMUK PV P 2051 from the Upper Cretaceous of Chalk Formation (Lewes, Sussex). (**a**) Photograph of the anterior portion of the specimen in right lateral view, including the abdominal cavity. (**b**) Three-dimensional reconstruction of the anterior portion of the specimen and the calcified lung. (**c-e**) Transverse sections of a high-resolution computerized axial tomography scan of NHMUK PV P 2051. Pink arrows pointing to the bony plates. Red, calcified lung; beige, skeleton and sediment. 1, 2, 3, pointing to the

successive areas of the section in respectively e, d, c. Numbered asterisks pointing to the lumen of the calcified lung. Scales bars, 5 cm (**a, b**); 1 cm (**c-e**).
